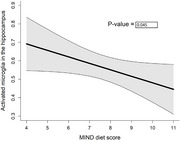# The MIND diet association with lower microglial inflammation in the hippocampus: A Community‐Based Neuropathologic Study

**DOI:** 10.1002/alz70860_100482

**Published:** 2025-12-23

**Authors:** Puja Agarwal, Alifiya Kapasi, Sonal Agrawal, Maude Wagner, Francine Grodstein, David A. A. Bennett, Lisa L. Barnes, Sue E. Leurgans, Julie A Schneider

**Affiliations:** ^1^ Rush Alzheimer's Disease Center, Rush University Medical Center, Chicago, IL, USA; ^2^ Rush University Medical Center, Chicago, IL, USA; ^3^ Department of Pathology, Rush University Medical Center, Chicago, IL, USA; ^4^ Department of Neurological Sciences, Rush University Medical Center, Chicago, IL, USA; ^5^ Department of Internal Medicine, Rush University Medical Center, Chicago, IL, USA; ^6^ Department of Psychiatry and Behavioral Sciences, Rush University Medical Center, Chicago, IL, USA; ^7^ Department of Neurology and Pathology, Rush University Medical Center, Chicago, IL, USA

## Abstract

**Background:**

Neuroinflammation is a risk factor for Alzheimer's Disease (AD) and cognitive decline in aging. Reactive microglia are the first response to neuroinflammation. In animal studies, long‐term exposure to a high‐fat diet enhances reactive microglia and cognitive impairment. Healthy diets, such as MIND (Mediterranean‐DASH Intervention for Neurodegenerative Delay), are rich in antioxidant nutrients and are associated with slower cognitive decline among older persons. However, the link between diets with anti‐inflammatory properties and microglial inflammation in human brains is unknown. This study examines the association of the MIND diet and antioxidant nutrients with microglia in the hippocampus.

**Methods:**

Analyses included 237 autopsied participants (age at death=91.2±6.1 years, 72% females, education = 15.3±2.8 years) from the Rush Memory and Aging Project, without known dementia, with hippocampal microglia data and complete dietary data over an average follow‐up of 7.2±4.4 years. The microglia burden in the mid‐hippocampus and anterior hippocampus was quantified using stereology. Based on cellular morphology, we counted the number of total microglia and reactive microglia (stages 2 and 3), which were defined as having hypertrophic/amoeboid morphology. Mean MIND diet scores (range=0‐15) and calorie‐adjusted antioxidant nutrients were computed from all food frequency questionnaires obtained during follow‐up. Linear regression models were used, and nutrients were assessed in quintiles.

**Results:**

Higher MIND diet scores during the follow‐up were associated with fewer reactive microglia (i.e., less neuroinflammation) in the hippocampus when controlled for age at death, sex, education, calories, and AD pathology (β=‐0.035, SE=0.02, *p* = 0.045), but not with total microglia (β=‐4.88, SE=3.08, *p* = 0.114). Among calorie‐adjusted antioxidant nutrients (models controlled for age at death, sex, education), oleic acid (mono‐unsaturated fatty acid) was negatively associated with activated hippocampal microglia (Q5 vs. Q1: β=‐0.18, SE=0.08, *p* = 0.03) and vitamin E, which approached but did not reach significance (Q5 vs. Q1:β=‐0.13, SE=0.07, *p* = 0.08).

**Conclusion:**

The MIND diet is associated with microglial inflammation in the hippocampus. Further studies are needed to investigate if the diet's role in lowering the overall neuroinflammatory response explains the association of diet and cognitive health in older persons. Antioxidant nutrient analysis indicates a potential inverse relation between dietary vitamin E and oleic acid with hippocampal microglia that warrants further investigation with bigger sample size.